# "Why I stay in community psychiatric rehabilitation": a semi-structured survey in persons with schizophrenia

**DOI:** 10.1186/s40359-022-00919-0

**Published:** 2022-09-06

**Authors:** Shan-Shan Zheng, Hui Zhang, Man-Hua Zhang, Xue Li, Kuo Chang, Feng-Chi Yang

**Affiliations:** 1 School of Medical Humanities, Capital Medical University, No. 10, Xitoutiao, You An Men Wai, Fengtai District, Beijing, 100069 China; 2 Community Health Service Center in West District of China Agricultural University, Courtyard 2, Yuan Ming Yuan West Road, Haidian District, Beijing, 100091 China

**Keywords:** Community, Qualitative research, Rehabilitation, Schizophrenia

## Abstract

**Aims:**

Although community psychiatric rehabilitation plays an important role in returning persons with schizophrenia to the society, many patients in China stay in rehabilitation centers for longer periods of time and subsequently fail to integrate. This study is aimed to explore the underlying causes of this trend and identify possible solutions.

**Methods:**

This study used a qualitative descriptive design to examine the persons with schizophrenia who stay in rehabilitation centers for longer periods of time. The researchers conducted semi-structured telephone interviews with the patients recruited through purposeful sampling. The audio-recorded interviews were transcribed in transcripts in Chinese. Thematic analysis was performed using Colaizzi's 7-step method.

**Results:**

Most patients believe that they have gained knowledge, improved skills, friendship and social circles through community mental rehabilitation, with the sense of belonging and enriched life strongly attracting them to the rehabilitation centers. They felt that the difficulty of further integration into society is mainly because of social prejudice and rejection. In addition, the activities of community mental rehabilitation meet the needs of social communication, which also hinder patients from further entering the society.

**Conclusions:**

Persons with schizophrenia with long-term stay in community mental rehabilitation centers meet their friendship, sense of belonging and social needs by participating in rehabilitation activities. Providing special social opportunity for these patients can get them out of the rehabilitation center. Overall, it is possible for patients to gradually return to society in a collective form.

## Background

Schizophrenia, a serious chronic mental illness that affects more than 20 million people in the world [1] seriously affects human health [2, 3], and exacerbates social [4] and economic [5] burden not only for patients, but also to their families, other caregivers in the society. Persons with schizophrenia encounter various obstacles in perception, thinking, emotion, behavior, and significant abnormalities in mental activities, which subsequently cause marked social impairment. Although drugs can control schizophrenic symptoms, they don't improve a patient’s social function [6]. Mental rehabilitation is also known as psychiatric rehabilitation, is the process for persons with chronic mental illness to restore community functioning and improve their quality of life. Consequently, many countries [7, 8] have incorporated rehabilitation into their national policies on mental health, as well as formulate service standards and guiding principles. Generally, the goal of mental rehabilitation is to help patients lead a meaningful life in their own community [9]. Most patients choose medication at home and community follow-up when their conditions are stable. To date, community-based mental rehabilitation is an important step for integration of these home-treated patients into both the family and society [10, 11].

Psychiatric rehabilitation in China started decades later than that in developed countries, while community-based rehabilitation has only developed in the past ten years. In the capital, Beijing, resources for rehabilitation are quite short, due to various reasons such as lack of experience, limited space, and shortage of staff, among others. At the same time, many patients who have been rehabilitated for several years and have improved social functions, still participate in rehabilitation activities every week. Notably, these patients use the available resources making it difficult for those with poor social function and who urgently need rehabilitation to join. Ensuring that patients with improved social functions leave rehabilitation centers is imperative to enable those in urgent need access the limited rehabilitation resources. Numerous studies have focused on community psychiatric rehabilitation, owing to its importance for patients. The most applied rehabilitation measures across communities include health education (disease knowledge and prevention of recurrence), physical exercises, group discussions, and trainings for cognitive, life, vocational and social [11–15]. The current developments in psychotherapy have resulted in new interventions, such as art therapy [15], mindfulness-based interventions [16, 17], meta cognitive training [12], and horticultural therapy [18], among others, and these are now extensively applied in the community. Moreover, evaluating the effect also extends from superficial symptoms, cognition ability and skills to deeper levels, such as psychological feelings and quality of life. Results from previous meta-analyses and pilot studies have demonstrated the effects of these rehabilitation efforts, including improvement of patients' symptoms [10, 18–20], increase in medication compliance [21, 22], improvement of life skills [23], improvement of mood [22], increases in self-esteem [24, 25], and improvement in quality of life [10, 11, 18, 24] among others. However, most of these studies represent researchers’ perspectives. Determining the perception of patients under rehabilitation is imperative to understanding the effect of this therapy. Some previous qualitative studies have evaluated the perceptions of patients and their caregivers and found that motivation needs for people under rehabilitation are different [26–28]. However, only a handful of these studies have described the long-term effects of rehabilitation in patients, which is common in China, possibly due to different national conditions. In addition, no study in China has focused on patients who are engaged in long-term rehabilitation activities.

To ensure that the limited resources in rehabilitation benefit more patients, it is necessary to solve the problem associated with long-term stays in rehabilitation by some patients. Understanding reasons why these patients stay for longer periods is imperative to designing accurate exit strategies for them and identification of alternative activities that satisfy them. In the present study, we interviewed persons with schizophrenia who have stayed in rehabilitation centers for many years with the aim of identifying the reasons why they have not integrated into the society. Our findings are expected to provide valuable insights to guide effective and applicable rehabilitation policies, which will in turn ensure that the limited rehabilitation resources can play a greater role in patient integration back to the society.

## Methods

### Design

We adopted a qualitative study design, based on semi-structured telephone interviews, to understand participants' personal experiences and feelings under rehabilitation. All interviews were conducted between April and June 2021. The interview guide was developed by the research team based on the study aims and available literature. Then it was adjusted according to pilot study and expert opinion. The main interview questions were as follows: (1) How do you think the community psychiatric-health rehabilitation helped you? (2) What has changed in your life after joining in rehabilitation? (3) What rehabilitation is your favorite and why? (4) What attract you in this group? (5) How long do you plan to take part in rehabilitation? (6) Do you join other social group? If yes, what? If no, why? (7) What difficulties have you met in further social activities?

### Participants and selection criteria

Purposeful sampling was used to recruit persons with schizophrenia in the study. Eligible participants, across 6 community psychiatric rehabilitation centers of Beijing Haidian district, were referred by psychiatrists. All participants were diagnosed with schizophrenia, which was confirmed by psychiatrists at the mental hospitals. Criteria for inclusion and exclusion of patients in the study are summarized in Table 1.

**Table 1:** Inclusion and exclusion criteria for persons with schizophrenia enrolled in the study

*Inclusion criteria*
1) Diagnosed with schizophrenia, by a psychiatrist, according to the ICD-10 guidelines
2) Had received community psychiatric rehabilitation for 5 or more years
3) Gave informed consent
4) Understood and communicated in Chinese
5) Were not diagnosed with any severe physical disease
*Exclusion criteria*
1) Had hearing or speech impairment
2) Were receiving any other psychiatric rehabilitation
3) Had serious cognitive impairment

ICD, International Classification of Diseases

### Data collection

This was an initial phase of a doctoral study titled "Effect of group cognitive behavior therapy on rehabilitation of patients with severe mental disorders". Ethical approval for the study was obtained from the Ethics Committee of Capital Medical University. The purpose and procedures of the study were explicitly explained to the participants, who then voluntarily signed a written consent prior to the interview. Since confidentiality was an important part of the research, we used numbers instead of names to ensure participant anonymity. Psychiatrists across the 6 community psychiatric rehabilitation centers provided demographic information for the recruited participants and obtained their consents. Subsequently, the first author (PhD candidate) arranged the interview time, and conducted all individual interviews via the telephone. All the participants were native speakers of Chinese, so the interviews were conducted in Chinese*.* The interviews were based on open-ended questions, with many examples and details collected to clarify the information. All the interviews were audio recorded, with each one taking between 25 and 43 min.

### Data analysis

The audio-recorded interviews with the patients were transcribed verbatim into word documents by the first author in Chinese. The documents were then checked for accuracy by two people who were not associated with the study. The data were extracted inductively from the interview transcripts. Summaries of codes and themes are presented in Table 2. For data analysis, we adopted Colaizzi's 7-step analysis method [29] for phenomenological data: (1) carefully read through interview records; (2) extract important and meaningful statements; (3) encode recurring and meaningful content; (4) collect encoded views; (5) write down detailed and exhaustive descriptions; (6) distinguish similar views and sublimate theme concepts; (7) return findings to the participants for verification of ambiguous information. The first and second authors independently coded the data, formed a coding framework through discussions, then extracted themes and subthemes for analysis in research team meetings. The research team included psychiatrists and psychiatric rehabilitation experts, which was an important aspect to enhance understanding and interpretation of the results. Many meetings were convened to discuss, define, and review themes until an agreement was reached.

**Table 2:** Themes, subthemes and codes

Themes	Subthemes	Codes
Rehabilitation effect	Knowledge and adherence (16/28)	Understand the symptoms Chronic illnesses Long-term use of medication Relapse prevention Confidence in disease control
	Improvements in living ability (14/28)	Self-care Doing housework Increase frequency of going out Get active about physical activity Levels of stigma reduced Emotional stability
	Friendship and social interaction (24/28)	Making friends Care about each other Mutual understanding Have a good time with each other No discrimination and stigma More social opportunity Good social place Maintaining social contacts
Attractiveness	Sense of belonging (24/28)	An equal and safe social environment Talk freely Enjoy being together Family-like group Feeling respected and loved Receive encouragement and support All my friends are here
	Enriched and interesting lives (25/28)	A variety of rehabilitation activities Learn many things Feel happy in rehabilitation activities Sense of accomplishment and self-worth
Difficulties in social communication	Social-environmental factors (27/28)	Social rejections, Discrimination and prejudice Lack of work opportunities
	Self-stigma (22/28)	Unconfident or low self esteem People might not accept me Staying away from other people Avoid conflict
	Lack of motivation (21/28)	Community rehabilitation is enough Satisfied with life

## Results

### Participant characteristics

A total of 32 patients were invited to participate in this study. However, 2 declined the invitation for no reason while another 2 failed to complete the invitation. Finally, 28 patients across 6 community rehabilitation centers were interviewed. The participants’ ages ranged from 35 to 67 (with a mean of approximately 50.5) years. Mean duration of illness of the schizophrenia was 22.71 years (SD = 6.72), and mean period of community rehabilitation was 9.43 years (SD = 1.83). All participants in this study lived at home, 22 (78.57%) were living with their families, 2 (7.14%) were living with relatives and 4 (14.29%) were living alone. They were receiving treatment with oral antipsychotic medication, with most patients being administered two or more drugs. A summary of their characteristics is outlined in Table 3.

**Table 3:** Participants' characteristics

Variable	Number	Proportions (%)
Persons with schizophrenia	28	
*Gender*		
Female	17	60.7
Male	11	39.3
*Education*		
Primary school	1	3.6
Junior high school	5	17.9
Senior high school	13	46.4
University or above	9	32.1
*Marital status*		
Married	12	42.9
Single or divorced	16	57.1
*Children*		
With children	13	46.4
Without children	15	53.6

### Qualitative analysis

The generated data were stratified into three main themes, namely effect of community rehabilitation, attractiveness, and difficulties in social communication.

**1. Effect of community rehabilitation**

Here, three main dimensions that focus on the effect of community rehabilitation, namely knowledgeable of disease and adherence to treatment, improvement in life ability as well as stable friendship and social interaction, were analyzed.

**1.1 Knowledge of the disease and adherence to treatment**

Among the 28 respondents, 16 stated that they had understood disease characteristics and the importance of adhering to medication more explicit because of various health educations. From the responses, all patients adhered to medication, although some said they had done well before joining rehabilitation. Knowledge from rehabilitation activities strengthened their belief, while more patients accepted the reality of their illness and the side effects associated with medicine rather than fear or avoidance. Some of their responses are as follows:

The doctor told me that I was not crazy, but sick. I couldn't control the performance of the illness. That was not my fault. I would be fine if I could take the medicine regularly. It is a chronic disease just like hypertension. (No.6).

I didn't know what the disease was at the beginning. I have learned more (from community rehabilitation) about it. (No.12)

I just can't sleep and think more about everything sometimes, now I know that’s also a symptom of my disease. (No.20)

In the past, I always stopped taking the drugs as soon as the symptoms improved. Now I know that I must take the medicine consistently to prevent a recurrence. I will never stop the medicine in the future. (No.24)

**1.2 Improvements of deficits in daily living ability**

A total of 14 correspondents mentioned effect of rehabilitation on improved living or coping ability. This was manifested across many aspects, such as taking care of oneself in life, taking care of family members, emotional processing, and social interaction. Some of their responses were as follows:

I used to respond very slowly. I needed to redial several times when I made a call, but now I do everything much faster. (No.9)

I used to be timid and hardly to go out. Now I go for a walk every day and I can help my family with shopping. (No.7)

In the past, I always felt that others were talking about me behind and thought that the loud voice from others was aimed at me. It's better now. I understand it has nothing to do with me whatever others say or do. (No.28)

In the past, I was very nervous and angry when I heard somebody talking about my illness. Now, I know that although I can't stop their talking, I can control my response. My emotions should not be influenced by others. (No.27)

**1.3 Stable friendship and social interaction**

24 out of the 28 respondents stated that they had forged friendships in rehabilitation, while 19 of them indicated that rehabilitation was the most important social activity of their current lives. Some of their responses were as follows:

We have been together (in the community psychiatric rehabilitation centers) for many years. Some of them (patients) are my friends. I missed them very much when the rehabilitation activities stopped because of the Covid-19 last year. (No.5)

Everyone here is in the same situation (with mental illness). No one laughs at me. I am willing to tell them something (that happened in my life). They will encourage me and give me some useful advice. (No.19)

At first, I thought that rehabilitation activities were just playing. Everyone treated me very well, and I learned new skills from the interesting activities. In short, I feel very happy here. Recently, I find that I can comfort and help others sometimes, which give me a sense of accomplishment. (No.11)

Apart from coming here (to participate in rehabilitation activities), I don't have any other activities with others. (No.25)

**2 Attractiveness**

Participants had been under rehabilitation for so many years that they wanted to continue until the rehabilitation center was not allowed due to age or health reasons. The main reason for rehabilitation attractiveness was due to a sense of belonging, and the associated enriched and interesting lives.

**2.1 Sense of belonging**

24 of the enrolled participants alluded to friendship, support and being supported, an equal and safe social environment, and a family-like group, as some of the reasons they felt a sense of belonging at their rehabilitation center. Some of their responses were as follows:

I had no friend since I got sick. I made friends again when I came here. It's not easy (to make these friends) for me, so I cherish (these friendships) very much. (No.2)

All my best friends are here. It's wonderful that we can talk and play together every week. Here (community psychiatric rehabilitation center) is another big family for me. (No.17)

I don't have any friends in my life. It's great being accepted by the (rehabilitation) center where I can open my minds to others, which is necessary for me. (No.26)

Moreover, 18 correspondents indicated that they had experienced respect and equality in their rehabilitation group. In fact, they found it easy to speak more freely because there was no discrimination. Some of their responses included:

We are all suffering from this disease (mental illness). We have a lot of common topics due to the similar experiences and feelings. Nobody looks down up on others. (No.1)

The doctors, nurses and teachers here (lectures or music, art, and handicrafts, among others) are very nice. If we could not understand or catch up, they would explain again and again with more patient than our family members. (No.23)

It's okay to make a joke here, since we understand each other. Joking is unimaginable in other social lives. (No.15)

I feel pretty good as long as we are together, no matter what rehabilitation activities to do. (No.4)

**2.2 Enriched and interesting lives**

Respondents with mental illness indicated that their daily lives were mostly monotonous and repetitive due to lack of work and social interaction. Specifically, 25 patients out of the 28 enrolled participants indicated that various activities organized by the rehabilitation centers made their lives rich and interesting. Some of their responses were:

I come here (to participate in rehabilitation activities) every week, otherwise I am watching TV or mobile phone at home, which is very boring. (No.22)

The rehabilitation activities include making handicrafts, paintings, planting, arranging flowers, and picking fruits among others, nearly all of which can’t be done in the family. That's why I like to be here. (No.14)

I like singing. The rehabilitation center often organizes rehearsal programs to participate in performances during festivals. I am always the lead singer in the chorus show, which give me a sense of accomplishment. (No.3)

Those of us who have recovered have the opportunity to travel nearby. It's great to spend a day in the scenic spot and have lunch in a restaurant. This makes our relationship closer. (No.26)

**3. Difficulties in social communication**

Participant responses revealed existence of 3 difficulties under rehabilitation, namely not being accepted by society, self-stigma and low self-confidence, and lack of motivation and interest.

**3.1 Not being accepted by the society**

Nearly all respondents strongly believed that they were excluded from society and had experienced social rejections. Specific shows include discrimination and prejudice, sarcasm and ridicule, social isolation and lack of job opportunities.

After all, we are different from the normal persons. It’s better to stay away from them rather than they avoid and laugh at me. (No.21).

Even my family members don't like me, let alone strangers. (No.19)

I know other people have looked down on me and call me a lunatic. (No.13)

Nobody wants to hire people like us (persons with schizophrenia). Some shopkeepers even prevent me to enter the store, for fear of affecting their business. (No.8)

**3.2 Self-stigma and low self-confidence**

Patients' self-stigma and low self-confidence were mainly displayed in self-isolation during daily life. Notably, the correspondents felt they didn't strive for themselves even during interactions with others. Specifically, 12 correspondents avoided communicating with others when outside, 4 had jobs in supportive employment where they obeyed the arrangement and never asked for any needs, while 15 selected concessions when faced with conflicts with others in public. Some of their responses included:

I go out every day to exercise, all by myself, and try to keep away from the crowd. (No.11).

This disease (schizophrenia) is rather special, no one is willing to socialize with people with this disease. So, I'd rather stay alone, lest others disgust me. (No.14)

I have a job in the sub-district, mainly responsible for cleaning. I was willing to take on more works, but I didn't mention, for fear that I would lose this job. (No.16)

There are also some group activities in my community, I never try to participate in because I am sure that they must mind (that I have schizophrenia). (No.3)

I never quarrel with people outside. I am always the first one to make a concession, even though I am right. What I fear mostly is (the sentence) 'he is sick/crazy' and (this phrase) nearly can be heard every day. (No.10)

**3.3 Lack of motivation and interest**

Almost all patients indicated that rehabilitation was one of their most important social activities, with more than 20 participants indicating that rehabilitation was their only external social activity as well as the only means of communication with relatives. Some of the responses included:

The society is too complicated for me. The community rehabilitation brings me happiness and hope. (No.18)

Anyway, as long as I have time, I will continue to participate in the (rehabilitation) activity here. I don't want to participate any other groups. (No.2)

Friends and activities here are enough, I don't need any more. (No.23)

It's enough to participate in activities here every week, and more activities will make me too tired. (No.17)

## Discussion

The present survey reveals the perceptions of persons with schizophrenia who have spent long periods of time under rehabilitation. From the collected information, three main information subthemes, namely reasons why the patients were willing to join rehabilitation, benefits of engaging in the activities, and the obstacles they face during transition from the rehabilitation centers to the real society, emerged. Specifically, the respondents indicated that rehabilitation activities had markedly improved their medication compliance, life skills and coping ability. Interestingly, the patients believed that friendship, a sense of personal belonging and value gained under rehabilitation were more important than the aforementioned gains. Various rehabilitation activities had enriched the patient's daily life. However, they hindered them from further integration into society to some extent. These results, which are based on patients' experience, can help us to comprehensively understand the current status of rehabilitation for this group of patients. These findings are expected to guide future designs into effective approaches for seamless integration of these patients into society.

### Knowledge and ability gains

The findings of this study indicated that rehabilitation enhances patients' ability to learn more about the disease, improves their medication adherence, boosts their skills such as living ability, social interaction and problem solving. Information regarding the signs and symptoms of schizophrenia, as well as the reasons for relapse is important for patients. This is because once they not only understand that the disease causes many problems, but they are also gain deeper insights into drug efficacy, phenomena that consequently promote their adherence to medication as previously reported [22, 30]. Apart from the psychiatrist’s guidance, members of the rehabilitation group usually exchange knowledge regarding the effects and side effects of medication. Doctors, rehabilitation staff, other patients and family members represent an important social support system for persons with schizophrenia. Previous studies have shown that good social support can increase compliance [31]. Other evidences have demonstrated that the most important treatment approach for controlling schizophrenic symptoms is medication, which can significantly reduce the recurrence and re-hospitalization rates in patients [32, 33]. In addition, improvement of living standards and social ability are closely associated with targeted training during rehabilitation, which also represents one of the most important activities of early stage rehabilitation. Specifically, improvement of patients' problem-solving ability correlated with improved life ability and social interaction. Therefore, community psychiatric rehabilitation centers have proved to be relatively safe places that provide an equal environment for patients to continually practice the skills they learned, thereby improving their problem-solving ability. This is consistent with findings from previous systematic studies [16, 18].

### Benefits of friendship, and a sense of personal belonging and value

Patient responses indicated that friendship and fixed social activities are very important benefits of rehabilitation. Specifically, most patients believe that friendship and social interaction have changed their lives, from boring to interesting, and from monotonous to diverse. In fact, the positive energy they have gained gives them full hope for the future. Moreover, they also indicated that community psychiatric rehabilitation centers have given them a sense of belonging. Previous studies have shown that having hope and feeling a sense of belonging are important aspects for improving quality of life [34, 35]. The factors that are mainly considered include improvement of cognition [36], mood [22], skills [23], while friendship and hope are rarely mentioned. In the present study, respondents indicated that they had higher friendships than other improvements, with someone even indicating that friendship was the most important gain. Previous studies have also shown that friendships made under community psychiatric rehabilitation centers are an important social support for patients, while social support is an important factor affecting the quality of life [37, 38].

### Reasons for patients’ willingness to participate in rehabilitation activities

This study found that some patients who had recovered for many years no longer benefited from rehabilitation activities, but they still actively participated in rehabilitation and didn't want to leave the rehabilitation group. The most important reason was that they valued the friendships of their group members and were unwilling to lose them. Persons with schizophrenia always isolate themselves and limit their scope of activities due to strong stigma. They lack friends and have a strong sense of loneliness [39]. However, members of the rehabilitation group meet their desire for friendship, and they must continue to participate in activities to consolidate the friendship. Participating in community rehabilitation activities may assist patients in finding new social roles. These can also help in reducing self-stigma and increasing the chances of reintegration into the real world [40]. We speculate that rehabilitation groups, unlike real communities, provide a more comfortable environment free of discrimination, avoidance, and rejection. Therefore, patients don't need to hide and disguise themselves while in rehabilitation centers. Patients in the groups respect, support, and encourage each other, giving them a great sense of happiness and value. All of the aforementioned benefits provide patients with a strong sense of belonging, which is difficult to get in the real society. Their emphasis on and pursuit of belonging has resulted in their high dependence on the rehabilitation group as the status quo. Understanding the reasons enables us to develop new group activities for patients, which can give them the sense of worth and belonging. Patients should be transferred from community psychiatric rehabilitation centers to new groups, leaving the limited rehabilitation to patients who need it the most urgently.

The study also found that rehabilitation activities enriched the quality of life for patients. Daily entertainment for patients is relatively simple at home, where they watch TV, use their mobile phones or go out to exercise alone. On the contrary, rehabilitation activities are diverse and interesting, introducing new experiences to the life of patients. The satisfaction and happiness gained from rehabilitation activities are often greater than those in daily life. Patients are full of expectations for the ongoing rehabilitation activities. To some extent, the expectation has become one of the goals of life for the patients, making it easier for them to cope with daily life.

### Difficulties in assimilating into real society

The main obstacles for patients to participate in general social activities are social prejudice and non-acceptance. There have been many previous studies on this subject [25]. The needs for wider social activities for patients have declined significantly because of the social interaction in rehabilitation groups, which has also become an obstacle for the patients. Although previous studies mentioned various benefits of rehabilitation groups [36, 41], they overlooked the fact that this benefit is also a hindrance. Only a few of the patients in this study expressed an interest in other social activities and never participated in them for various reasons. Although most patients expressed no interest in other social activities, it was clear from their attitudes towards rehabilitation that they had a strong interest in social events [42]. Participating in community rehabilitation activities may assist patients in finding new social roles to integrate into the society.. Previous studies proved the need for social interaction for patients. Patients may hide their social needs as a form of self-protection. For self-comfort, patients use "I don't need" rather than "I can't get". Similar to the results in another study, patients mentioned that a lack of friends was not a problem but rather an adaptive behavior [43]. The experience of stigma-based rejection, as well as current social discrimination and exclusion, may perpetuate their negative beliefs about social interactions [42].

Based on an analysis of the basic data of patients who have been in rehabilitation for a long time, we discovered that these patients have a longer duration and a fixed income. Most of them live with their families, which means that they have strong family support. According to Maslow's needs theory, these patients have met the two levels of physiology and safety, so the next goals are the needs for belonging and respect. Because most patients have a sense of family belonging, the need for friendship and social belonging is more urgent and the rehabilitation center simply meets their demand. Previous investigations on the friendships of schizophrenia patients had shown that although the friendship network of the patients was small, the quality of friendship was mostly positive, and patients attached great importance to friendship [43]. Reciprocal relationships between friends are essential for patients to improve response abilities and overall quality of life [44]. A survey on the needs of patients and caregivers showed that personal needs and emotional support (friends, partners, and family members) are the most important to mental patients [45].

Although extensive publicity and education has been done, the general public still knows little about mental illness, and the persons with schizophrenia also subject to high rate of stigmatisation and discrimination [46]. In such an environment, the self-stigma of the patient is aroused. Social stigma and self-stigma cause patients to habitually return to their safe zone (family and rehabilitation center) and avoid the real society. Job opportunities for patients with mental illness benefit from the supportive employment policy of the government, making patients to highly value job opportunities. Because the colleagues in a work environment are normal people, patients often lack self-confidence and fail to pursue more opportunities or resources. Besides explaining disease-related knowledge and skill training (such as daily living skills, social skills, and life skills), the rehabilitation centers provide various sports exercises, artistic treatments, and outing entertainment. The organizers intend for these activities to be used for skill training and social practice. However, for long-term recovery patients, these treatments, entertainment, and social functions are more critical than rehabilitation. Regular rehabilitation programs meet their basic social needs. In summary, routine family life and participation in rehabilitation have become a fixed life pattern for many patients because of the social exclusion and their low demand for social contact.

### Reflection and prospects

Understand how patients evaluate rehabilitation and why they stay in the rehabilitation centers can help rehabilitation managers to find solutions to the problem of patients overstaying in the rehabilitation centers. Because psychiatric rehabilitation is a developing field in China, the professional psychiatric rehabilitation providers are not enough. In this study, staff from the Beijing Disabled Persons' Federation and community psychiatrists or nurses collaborated to provide rehabilitation. In addition to rehabilitation work, the community psychiatrists or nurses have many daily management responsibilities, which limits the amount of time available for psychiatric rehabilitation. Every new patient who enters a rehabilitation group needs a series assessment of disease conditions and safety, which require more time from psychiatrists or nurses. Patients continue to participate in community psychiatric rehabilitation, reducing not only the workload of psychiatrists, but also the risk during rehabilitation activities. In the past, this state was suitable because many patients refused to participate in rehabilitation because the benefits of rehabilitation were not well-known, resulting in an oversupply of psychiatric rehabilitation. Recently, the abilities of the patients who joined rehabilitation gradually improved, and they approved the rehabilitation effect and were willing to continue rehabilitation. Because of the good curative effect, an increasing number of patients have been asked to participate in rehabilitation. However, due to staff and space constraints, the rehabilitation centers are unable to meet the rehabilitation needs of patients. Therefore, it is necessary to find suitable outlets for patients who are still stranded in rehabilitation centers after their life and social abilities have improved in order to provide rehabilitation opportunities to more patients with severely damaged social functioning. Rehabilitation is a long-term course that is required at every step from inpatient to outpatient and from returning to family to returning to society. Patients should be classified according to their remnant abilities, and treated with different rehabilitation measures, which requires a significant increase in the number of mental rehabilitation practitioners. Patients who are stranded in the rehabilitation centers have completed the rehabilitation course, and rehabilitation activities are important to meeting the needs of social interactions, the sense of belonging, and value. Development of other social groups and group activities that differ from rehabilitation centers in order to meet their needs maybe a feasible measure. Social workers or volunteers should organize cultural, artist, sports, or other entertainment regularly for the patients only, who can participate in any activity according to their interests. If possible, the government can also provide jobs in the community for well-functioning mental patients. The patients integrate into society gradually as a small group just like in the community rehabilitation centers.

### Limitations

This study used purposive and convenience sampling because it was concerned with long-term schizophrenia patients who wanted to participate in rehabilitation. Despite being sampled from multiple communities, all patients belonged to the same area, making it difficult to avoid selective migration. Respondents were voluntary patients, suggesting that they represent patients with the best social function rather than all patients who joined in the long-term rehabilitation. Furthermore, because the interviewer was a psychiatrist, power differentials may have affected negative information expression from the patient, resulting in information bias. Because telephone interviews may lose implicit information in facial expressions, we increased the sample size by 30% after reaching information saturation.

## Conclusion

This is the first study in China on schizophrenia patients who continue to participate in community psychiatric rehabilitation after their life and social abilities have improved. According to the results, community psychiatric rehabilitation improves life skills and social skills, but the patients believe that friendship, personal belonging, and a sense of self-worth are more important and attractive to them. Various types of rehabilitation activities enrich daily life and meet the social and entertainment needs of the patients. However, it has become an obstacle for patients to further integrate into society. Therefore, we can try to organize other group activities specifically who have recovered from mental illness and have good social functioning. These activities should not be aimed at functional recovery, but rather at meeting the social and belonging needs of the patients. Moreover, the government may provide these patients with collective jobs in order for them to gradually integrate into the society as a unit, which may be easier than as individuals.

## Data Availability

The datasets generated during the current study are not publicly available due participant anonymity, but are available from the corresponding author on reasonable request.

## References

[1] World Health Organization. Mental health action plan 2013–2020. 2013. Available from: http://www.who.int/mental_health/publications/action_plan/en/. Accessed 17 Oct 2021.

[2] Dieset I, Andreassen OA, Haukvik UK (2016). Somatic comorbidity in schizophrenia: some possible biological mechanisms across the life span. Schizophr Bull.

[3] Nishanth KN, Chadda RK, Sood M, Biswas A, Lakshmy R (2017). Physical comorbidity in schizophrenia & its correlates. Indian J Med Res.

[4] Charlson FJ, Ferrari AJ, Santomauro DF, Diminic S, Stockings E, Scott JG, McGrath JJ, Whiteford HA (2018). Global epidemiology and burden of schizophrenia: findings from the global burden of disease study 2016. Schizophr Bull.

[5] Chong HY, Teoh SL, Wu DB-C, Kotirum S, Chiou C-F, Chaiyakunapruk N (2016). Global economic burden of schizophrenia: a systematic review. Neuropsychiatr Dis Treat.

[6] Schennach-Wolff R, Jäger M, Seemüller F, Obermeier M, Messer T, Laux G, Pfeiffer H (2009). Defining and predicting functional outcome in schizophrenia and schizophrenia spectrum disorders. Schizophr Res.

[7] Bond GR, Drake RE (2017). New directions for psychiatric rehabilitation in the USA. Epidemiol Psychiatr Sci.

[8] Bhugra D, Pathare S, Joshi R, Ventriglio A (2018). Mental health policies in Commonwealth countries. World Psychiatry.

[9] Farkas M, Gagne C, Anthony W, Chamberlin J (2005). Implementing recovery orientated evidence-based programmes: identifying the critical dimensions. Community Ment Health J.

[10] Chen Y, Yau E, Lam C, Deng H, Weng Y, Liu T, Mo X (2020). A 6-month randomized controlled pilot study on the effects of the clubhouse model of psychosocial rehabilitation with chinese individuals with schizophrenia. Adm Policy Ment Health.

[11] Saha S, Chauhan A, Buch B, Makwana S, Vikar S, Kotwani P, Pandya A (2020). Psychosocial rehabilitation of people living with mental illness: lessons learned from community-based psychiatric rehabilitation centres in Gujarat. J Family Med Prim Care..

[12] Chen Q, Sang Y, Ren L, Wu J, Chen Y, Zheng M, Bian G, Sun H (2021). Meta cognitive training: a useful complement to community-based rehabilitation for schizophrenia patients in China. BMC Psychiatry.

[13] Killaspy H, Baird G, Bromham N, Bennett A (2021). Rehabilitation for adults with complex psychosis: summary of NICE guidance. BMJ.

[14] Biondo J, Bryl K (2020). When words aren't enough: dance/movement therapy and schizophrenia. Schizophr Bull.

[15] Lim E, Lee S-Y, Bahk W-M, Yoon B, Jon D-I, Kim MD, Park S-Y, Song M-K (2018). The effects of group integrative arts therapy based on social skill training on the social adaptive function, empowerment and subjective well-being in inpatients with chronic schizophrenia. Schizophr Bull.

[16] Hodann-Caudevilla RM, Díaz-Silveira C, Burgos-Julián FA, Santed MA (2020). Mindfulness-based interventions for people with schizophrenia: a systematic review and meta-analysis. Int J Environ Res Public Health.

[17] Jansen JE, Gleeson J, Bendall S, Rice S, Alvarez-Jimenez M (2020). Acceptance-and mindfulness-based interventions for persons with psychosis: a systematic review and meta-analysis. Schizophr Res.

[18] Lu S, Zhao Y, Liu J, Xu F, Wang Z (2021). Effectiveness of horticultural therapy in people with schizophrenia: a systematic review and meta-analysis. Int J Environ Res Public Health.

[19] Lutgens D, Gariepy G, Malla A (2017). Psychological and psychosocial interventions for negative symptoms in psychosis: systematic review and meta-analysis. Br J Psych.

[20] Ozdemir L, Safak Y, Orsel S, Kahilogullari AK, Karadag H (2017). Investigation of the efficacy of a psychiatric-social rehabilitation performed In patients with schizophrenia in a community mental health center: controlled study. Alpha Psychiatry.

[21] Üstün G, Küçük L, Buzlu S (2018). Identifying the schizophrenia patients attending the rehabilitation program conducted in Community Mental Health Centers in terms of some demographic variables, characteristics related to the ailment, adaptation to the treatment and self-efficacies. J Psychiatr Nurs.

[22] Alizioti A, Lyrakos G (2021). Measuring the effectiveness of psycho education on adherence, depression, anxiety and stress among patients with diagnosis of schizophrenia. A control trial Curr Psychol.

[23] Pan L, Mellor D, McCabe M, Hil B, Tan W, Xu Y (2011). An evaluation of the Shanghai Mental Health Service schizophrenia rehabilitation program. Am J Psychiatr Rehabil.

[24] Asher L, Hanlon C, Birhane R, Habtamu A, Eaton J, Weiss HA, Patel V, Fekadu A, De Silva M (2018). Community-based rehabilitation intervention for people with schizophrenia in Ethiopia (RISE): a 12 month mixed methods pilot study. BMC Psychiatry.

[25] Caqueo-Urízar A, Urzúa A, Habib J, Loundou A, Boucekine M, Boyer L, Fond G (2019). Relationships between social stigma, stigma experience and self-stigma and impaired quality of life in schizophrenia across three Latin-American countries. Eur Arch Psychiatry Clin Neurosci.

[26] Oyelade OO, Nkosi-Mafutha NG (2021). Living beyond the limitation: Rehabilitation, life and productivity of individuals with schizophrenia in South-West Nigeria. Health Expect.

[27] Chen L, Zhao Y, Tang J, Jin G, Liu Y, Zhao X, Chen C, Lu X (2019). The burden, support and needs of primary family caregivers of people experiencing schizophrenia in Beijing communities: a qualitative study. BMC Psychiatry.

[28] Parker S, Dark F, Newman E, Hanley D, McKinlay W, Meurk C (2017). Consumers' understanding and expectations of a community-based recovery-oriented mental health rehabilitation unit: a pragmatic grounded theory analysis. Epidemiol Psychiatr Sci.

[29] Abalos EE, Rivera RY, Locsin RC, Schoenhofer SO (2016). Husserlian phenomenology and Colaizzi’s method of data analysis: exemplar in qualitative nursing inquiry using nursing as caring theory. Int J Hum Caring.

[30] Eticha T, Teklu A, Ali D, Solomon G, Alemayehu A (2015). Factors associated with medication adherence among patients with schizophrenia in Mekelle, Northern Ethiopia. PLoS ONE.

[31] Torras MG, Tomàs EP (2018). Interventions to improve therapeutic adherence in subjects with schizophrenia. Papeles del Psicologo.

[32] Stubbs B, Firth J, Berry A, Schuch FB, Rosenbaum S, Gaughran F, Veronesse N, Williams J, Craig T, Yung AR, Vancampfort D (2016). How much physical activity do people with schizophrenia engage in? A systematic review, comparative meta-analysis and meta-regression. Schizophr Res.

[33] Krieger I, Bitan DT, Sharon-Garty R, Baloush-Kleinman V, Zamir L (2020). The effect of community-based mental health rehabilitation services for schizophrenia: a retrospective cohort study. Psychiatr Q.

[34] Işık I, Ergü G (2020). Hope and belonging in patients with schizophrenia: a phenomenological study. Perspect Psychiatr Care.

[35] Barut JK, Dietrich MS, Zanoni PA, Ridner SH (2016). Sense of belonging and hope in the lives of persons with schizophrenia. Arch Psychiatr Nurs.

[36] Varga E, Endre S, Bugya T, Tnyi T, Herold R (2018). Community-based psychosocial treatment has an impact on social processing and functional outcome in schizophrenia. Front Psychiatry.

[37] Lin SP, Liu CY, Yang CY (2018). Relationship between lifestyles that promote health and quality of life in patients with chronic schizophrenia: a cross-sectional study. Hu Li Yan Jiu.

[38] Prabhakaran S, Nagarajan P, Varadharajan N, Menon V (2021). Relationship between quality of life and social support among patients with schizophrenia and bipolar disorder: a cross-sectional study. J Psychosoc Rehabil Ment Health.

[39] Yildirim T, Kavak BF (2020). The relationship between internalized stigma and loneliness in patients with schizophrenia. Perspect Psychiatr Care.

[40] Abdisa E, Fekadu G, Girma S, Shibiru T, Tilahun T, Mohamed H, Wakgari A, Takele A, Abebe M, Tsegaye R (2020). Self-stigma and medication adherence among patients with mental illness treated at Jimma University Medical Center, Southwest Ethiopia. Int J Ment Health Syst.

[41] Drake RE (2017). The future of psychiatric rehabilitation. Epidemiol Psychiatr Sci.

[42] Weittenhiller LP, Mikhail ME, Mote J, Campellone TR, Kring AM (2021). What gets in the way of social engagement in schizophrenia?. World J Psychiatry.

[43] Harley EW-Y, Boardman J, Craig T (2012). Friendship in people with schizophrenia: a survey. Soc Psychiatry Psychiatr Epidemiol.

[44] Boydell KM, Gladstone BM, Crawford ES (2002). The dialectic of friendship for people with psychiatric disabilities. Psychiatr Rehabil J.

[45] Lahera G, Cid J, Gonzalez-Pinto A, Cabrera A, Mariner C, Vieta E, Arango C, Crespo-Facorro B (2020). Needs of people with psychosis and their caregivers: "In their own voice". Rev Psiquiatr Salud Ment (Engl Ed).

[46] Ran MS, Hall BJ, Su TT, Prawira B, Breth-Petersen M, Li X-H, Zhang T-M (2021). Stigma of mental illness and cultural factors in Pacific Rim region: a systematic review. BMC Psychiatry.

